# FFF-based high-throughput sequence shortlisting to support the development of aptamer-based analytical strategies

**DOI:** 10.1007/s00216-022-03971-2

**Published:** 2022-02-18

**Authors:** Valentina Marassi, Monica Mattarozzi, Lorenzo Toma, Stefano Giordani, Luca Ronda, Barbara Roda, Andrea Zattoni, Pierluigi Reschiglian, Maria Careri

**Affiliations:** 1 Department of Chemistry, University of Bologna, Via Selmi 2, Bologna, Italy; 2 byFlow Srl, Bologna, Italy; 3 Department of Chemistry, Life Sciences and Environmental Sustainability, University of Parma, Parco Area delle Scienze 17/A, 43124 Parma, Italy; 4 Department of Medicine and Surgery, University of Parma, Parco Area delle Scienze, 23/A, 43124 Parma, Italy; 5 Institute of Biophysics, CNR, 56124 Pisa, Italy

**Keywords:** Field-flow fractionation, Aptamers, Lysozyme, Screening method, Aptamer selectivity

## Abstract

Aptamers are biomimetic receptors that are increasingly exploited for the development of optical and electrochemical aptasensors. They are selected in vitro by the SELEX (Systematic Evolution of Ligands by Exponential Enrichment) procedure, but although they are promising recognition elements, for their reliable applicability for analytical purposes, one cannot ignore sample components that cause matrix effects. This particularly applies when different SELEX-selected aptamers and related truncated sequences are available for a certain target, and the choice of the aptamer should be driven by the specific downstream application. In this context, the present work aimed at investigating the potentialities of asymmetrical flow field-flow fractionation (AF4) with UV detection for the development of a screening method of a large number of anti-lysozyme aptamers towards lysozyme, including randomized sequences and an interfering agent (serum albumin). The possibility to work in native conditions and selectively monitor the evolution of untagged aptamer signal as a result of aptamer-protein binding makes the devised method effective as a strategy for shortlisting the most promising aptamers both in terms of affinity and in terms of selectivity, to support subsequent development of aptamer-based analytical devices.

**Graphical abstract:**

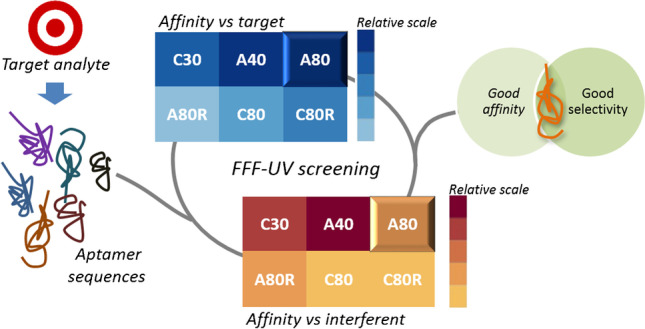

**Supplementary Information:**

The online version contains supplementary material available at 10.1007/s00216-022-03971-2.

## Introduction

The most recent evolutions in analytical chemistry involve the combination of miniaturized devices to proper biological recognition elements designed to interact with high affinity and selectivity with the target compound. The biorecognition elements can exploit naturally occurring interactions, such as antibody-antigen, enzyme–substrate, and DNA-DNA binding [1–3], or they can be biomimetic synthetic receptors such as molecularly imprinted polymer or aptamers [4–6]. Aptamers are short ssDNA or ssRNA sequences synthetized from nucleic acids as biological units and selected in vitro through the SELEX (Systematic Evolution of Ligands by Exponential Enrichment) iterative procedure, starting from an oligonucleotide library. These synthetic nucleic acids can fold into several three-dimensional structures as a function of their sequence and binding conditions; the interaction with the target is ascribable to a combination of electrostatic forces, hydrogen bonds, and π-π stacking.

Numerous techniques for the evaluation of aptamer-target binding are reported in the literature, i.e., surface plasmon resonance (SPR), size-exclusion chromatography (SEC), capillary electrophoresis (CE), isothermal titration calorimetry, affinity chromatography, and fluorescence spectroscopy, bearing in mind that the choice also depends on the dimension of the target compound, i.e., small molecules or proteins [7, 8].

Among the available techniques, separation techniques allow to directly study aptamer-target interactions and do not require immobilization, which can modify the nature and extent of the interaction [9]. SEC involves the separation of the species into a solid-phase packed column: non-native elution conditions can modify the aptamer structure with the risk of inducing artifacts. On the other hand, CE uses an open channel with an inevitable Joule heating when electric currents are passing through highly concentrated electrolyte solutions, thus affecting electrophoretic mobility and separation [10, 11]; in addition, CE has low sample capability. It should be noted that the affinity and selectivity of the interaction should be investigated in native conditions able to simulate the ionic strength and pH of the real matrix extract and the presence of possible interfering sample constituents. This necessity has led to the exploration of the use of asymmetrical flow field-flow fractionation (AF4) [11–13]. This separation technique is particularly suited for the analysis of samples in native or physiological conditions and for detecting aggregation, complexation, and conformational changes [14–16]. Following separation, AF4 can be used in combination with a variety of detectors, such as UV–Vis spectrophotometers and fluorimeters, ICP-MS, and laser scattering depending on the information required [17, 18]. In AF4, the mobile phase can be easily modulated to match the effective binding conditions, in which the bioreceptor-target interaction needs to be investigated, with the possibility to explore a wide range of ionic strength values maintaining native folding structure. In addition, AF4 is sufficiently versatile in terms of injection volumes, chosen on the basis of the downstream detection and applications (e.g., fraction collection).

Notwithstanding the potential and advantages of AF4, at now very few articles deal with the development of an AF4-based approach to investigate aptamer-protein interactions [11, 19]. In these studies, AF4 with fluorescence detection (FLD) has been used to monitor the binding between the fluorescently labeled aptamer and the target protein (IgE and streptavidin) and the isolation of an aptamer-protein complex. Ashby et al. have found that AF4 preserved the conformation of the protein and the protein-aptamer complex in solution, pointing out a discrepancy between the dissociation constant (*K*_d_) value obtained by AF4 and the previously published values for an anti-IgE aptamer: this can be attributed to the addition of a fluorophore to the short aptamer which can hinder the aptamer-protein binding interaction [11]. AF4 has also been coupled to SELEX enrichment for the selection of anti-DNA methyltransferase-1 aptamers, useful for therapeutic approaches [13]. All these studies were focused on aptamer-protein systems where the target protein had a much higher molecular weight than the aptamer: this condition favors a baseline separation of the peaks of the bound and unbound species in the fractogram, whose areas can be used for affinity assessment and calculation of the dissociation constant.

Among the already exploited aptamer-protein systems, there is an urgent need to develop a reliable screening strategy that could help to easily and quickly select, among sequences reported in literature, the most suited for a particular analytical application. The strategy should consider the constituents in the sample matrix, the possible interferents, and the necessity of sequence functionalization with tags for subsequent transduction.

Considering egg white lysozyme (14 kDa), different DNA aptamers have been exploited for the development of aptasensors for its determination in different food and biological matrices [20]. However, for the development of aptamer-based analytical methods, the choice of the aptamer sequence and binding conditions is commonly made regardless of the binding buffer used for SELEX selection or the downstream sample matrix. Phosphate-buffered saline and Tris buffers are the most exploited ones, but there is a high variability in terms of concentration, ionic strength, and presence or absence of Mg^2+^, which could play an essential role in aptamer folding [21–24].

In this context, the present work is aimed at moving a step forward to propose a fast AF4-approach able to pre-screen for the best aptamer candidate prior to the development of downstream analytical purposes, working on lysozyme-aptamer interaction, taking into account that the main difficulty for the investigated system relies in the very close molar mass of the aptamers to that of lysozyme. In addition, no fluorescent tags have been exploited. Our findings show that the use of specific absorption wavelengths can selectively detect the interaction between lysozyme and the evaluated aptamer without interferences and obtain information in less than an hour for each sequence tested. Finally, the same approach was taken to assess the selectivity of the same aptamers which were also incubated with bovine serum albumin, to understand the effect of non-specific interactions on aptamer applicability, and facilitate the selection of the most promising candidate.

## Materials and methods

### Chemicals

DNA sequences listed in Table 1 were purchased from Biomers.net (Ulm, Germany) in dried delivery state. Each lyophilized aptamer was dissolved in Milli-Q water to the recommended concentration of 100 μM and stored at – 20 °C. To avoid repeated freeze/thaw cycles, each stock solution was properly aliquoted. Egg white lysozyme and BSA were purchased from Sigma-Aldrich (Milan, Italy). The composition of the PBS-Mg^2+^ buffer (pH 7.4) used as mobile phase was the following: 1.5 mM KH_2_PO_4_, 8 mM Na_2_HPO_4_, 137 mM NaCl, 2.7 mM KCl, 1 mM MgCl_2_.

**Table 1:** DNA sequences

Name	Sequence	Molecular weight
A80	AGCAGCACAGAGGTCAGATG**GCAGCTAAGCAGGCGGCTCACAAAACCATTCGCATGCGGC**CCTATGCGTGCTACCGTGAA	24.7 kDa
A40	**GCAGCTAAGCAGGCGGCTCACAAAACCATTCGCATGCGGC**	12.3 kDa
A80R	AGCAGCACAGAGGTCAGATGACTATGTCGGCCGCAATGCCCAGAGGCCACATACAAGCGGCCTATGCGTGCTACCGTGAA	24.7 kDa
C80	*GGGAATGGATCCACATCTACGAATTC****ATCAGGGCTAAAGAGTGCAGAGTTACTTAG****TTCACTGCAGACTTGACGAAGCTT*	24.7 kDa
C30	***ATCAGGGCTAAAGAGTGCAGAGTTACTTAG***	9.3 kDa
C80R	*GGGAATGGATCCACATCTACGAATTC*AAGTGGATTAACTGTGTAGACCGATAGACG*TTCACTGCAGACTTGACGAAGCTT*	24.7 kDa

### AF4-UV analysis

The AF4-UV analyses were performed by using a 1100 Series HPLC system (Agilent Technologies, Palo Alto, CA), connected to a module to control AF4 flow rates and operations (Eclipse 3, Wyatt Technology Europe, Dernbach, Germany). Carrier solutions were degassed using an online vacuum degasser (Agilent, 1100 series, Agilent Technologies). Online detection of the eluted species was performed with an Agilent 1100 DAD UV/Vis spectrophotometer,

The AF4 channel was 152 mm long (Mini Channel, Wyatt Technology Europe), equipped with a regenerated cellulose membrane (Nadir), with a molecular weight cutoff of 5 kDa. The channel spacer was 350 μm thick. The injection volume was 16 μL.

The flow rate program was set up as follows: inject flow of 0.2 mL/min, a focus flowrate of 2 mL/min, a focusing time of 3.5 min, an initial cross-flow of 2.5 mL/min decreasing to zero in 5 min, and a detector flow of 0.4 mL/min. The total method duration was 11.5 min. PBS-Mg^2+^ binding buffer was used as elution medium.

The recovery of aptamers and proteins in AF4 channel was calculated as the % ratio between the signals obtained in focus-flow-injection analysis (Focus-FIA) and in flow-injection analysis (FIA), i.e., % Focus-FIA/FIA. In fact, in FIA the cross/focus flow is absent; thus, there is no interactions with the membrane that could cause sample loss, differently from Focus-FIA when the sample is subjected to a cross-flow.

Method precision was assessed both on retention times and on signal intensity by performing three independent replicates for each protein and aptamer. The same number of replicates (*n* = 3) were carried out for the analysis of each aptamer to protein ratio. The limit of quantification (LOQ) was calculated as the injected amount (pmol) at which the signal is 10 times the signal noise of the baseline. Linearity was verified by Mandel’s fitting test within the concentration range explored in binding experiments. All graphs were elaborated in GraphPad Prism.

### Preparation of aptamer-protein mixture

Aptamer-protein mixtures were prepared both for lysozyme and bovine serum albumin (BSA, potential interfering protein) to obtain molar ratios of 1:1, 1:2, 1:5, and 1:10. More precisely, 10 μL of 5 μM aptamer solution were mixed with 10 μL of solution of protein at different concentration levels (5, 10, 25, 50 μM). The solutions were prepared in PBS-Mg^2+^ buffer, the same used as mobile phase. For the analysis of aptamer alone, 10 μL of 5 μM aptamer solution were mixed with 10 μL of buffer.

## Results and discussion

### Aptamer sequences under investigation

The anti-lysozyme aptamers exploited in literature and adopted in this study are derived from two independent SELEX selections [20, 25, 26]: A80 and C80 are two full-length sequences, containing two fixed primer sequences (flanking regions), one on each side of a central domain that is randomized; A40 and C30 are derived from A80 and C80, respectively, by removing the primers. A80R and C80R are DNA oligonucleotides used as negative control: in particular, they are derived from randomization of the central region of A80 and C80, respectively, in order to simulate the randomization process involved in the library construction during SELEX process.

### Development of the AF4-UV method

The development of the AF4-UV method first involved the selection of the channel membrane: for this purpose, a polyethersulfone (PES) membrane and a regenerated cellulose (RC) membrane, both with a molecular weight cutoff (MWCO) of 5 kDa, were compared in terms of recovery values, calculated in triplicate. While all the aptamers and BSA showed a good recovery in both membranes (> 98% for aptamers and > 99% BSA), recovery for lysozyme was good in RC (> 97%), whereas a value of 75% was obtained when the PES membrane was used. The latter result can be due to interactions between the sulfone groups in PES leading to electrostatic interactions with lysozyme. Consequently, the RC membrane was chosen for subsequent method development, ensuring a good recovery for all the investigated species. Focus flowrate in the 1.0–2.5 mL/min range was investigated, which was found not to influence recovery; then, a value of 2.0 mL/min for focus flowrate was chosen since it reduces system peaks due to changes in flow direction. The next step was the evaluation of the intensity and duration of the cross-flow, which was performed by evaluating retention and separation between lysozyme and BSA as a reference. A gradient method was chosen since it is more adequate to reduce band broadening of bigger species with respect to isocratic methods. In addition, taking into account that the aim of this study was to provide a rapid method to screen for a high number of aptamers and both for target and interfering compounds, it was also necessary to set up a detection method which could be applied to all combinations and independently from the presence of fluorescent tags.

**Fig. 1:**
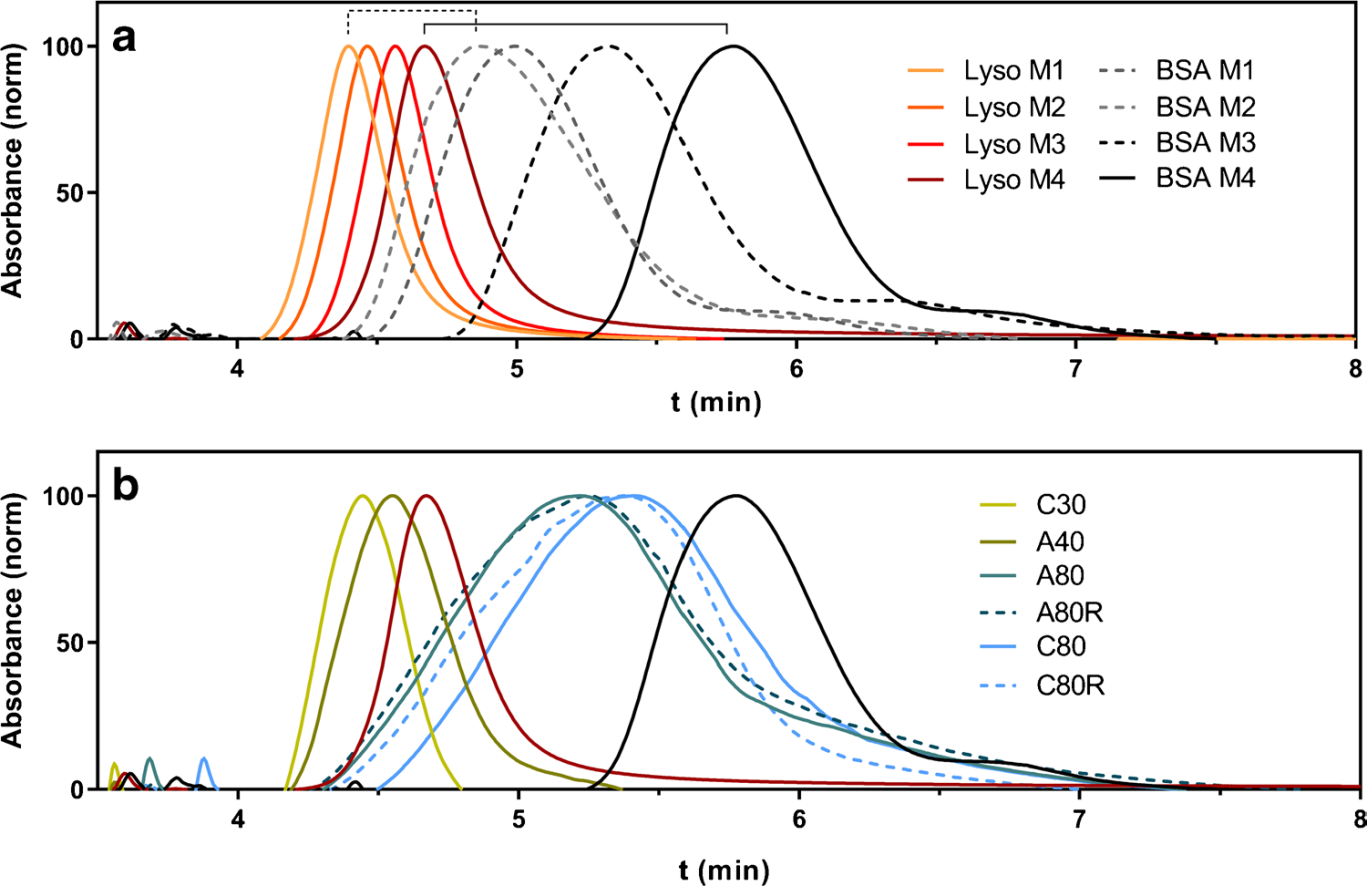
AF4-UV fractograms of **a** lysozyme (Lyso, orange to red traces) and BSA (grey to black traces) under varying method conditions. Solid lines: profiles obtained using the final method. **b** All aptamers using the final method. Red: lysozyme profile; black: BSA profile

The fractograms at different conditions are reported in Fig. 1a. After the focusing at 2.0 mL/min for 3.5 min, the starting cross-flow was initially set at 1.0 mL/min (method 1, M1), reaching zero in 4 min, which allowed the two proteins to differ in retention time by 0.5 min (Fig. 1a, dashed bracket). The initial cross-flow was progressively increased to 1.5 mL/min (M2) and to 2.5 mL/min (M3), which improved separation. Finally, the gradient duration was raised to 5 min, achieving a retention difference of more than 1 min (Fig. 1a, solid bracket) and an almost baseline separation (M4).

All the ssDNA sequences were analyzed under the established conditions to verify that all species could be eluted within 5 min of the method gradient after the focusing time (Fig. 1b).

Lysozyme and aptamer sequences have very similar molar mass (14 kDa for lysozyme and 9 to 25 kDa for aptamers), and they have similar retention times. As expected, smaller aptamers (C30, A40) had a lower retention time than lysozyme, while the others (A80 and A80R, C80 and C80R) had a higher retention time, though they were all eluted before BSA. Furthermore, it can be observed that all peaks, especially those relative to longer aptamer sequences, are very broad, which is compatible with the conformational freedom of aptamers in solution. The wide hydrodynamic radius distributions of the investigated species would hinder the measurement of size changes by conventional sizing batch techniques such as dynamic light scattering (DLS), considering also in this case the simultaneous presence of bound and unbound species.

The potential of the developed method is demonstrated by the reproducibility of peak intensity and retention times, which shows standard deviations less than 3% and 0.1 min for all the species, respectively.

**Fig. 2:**
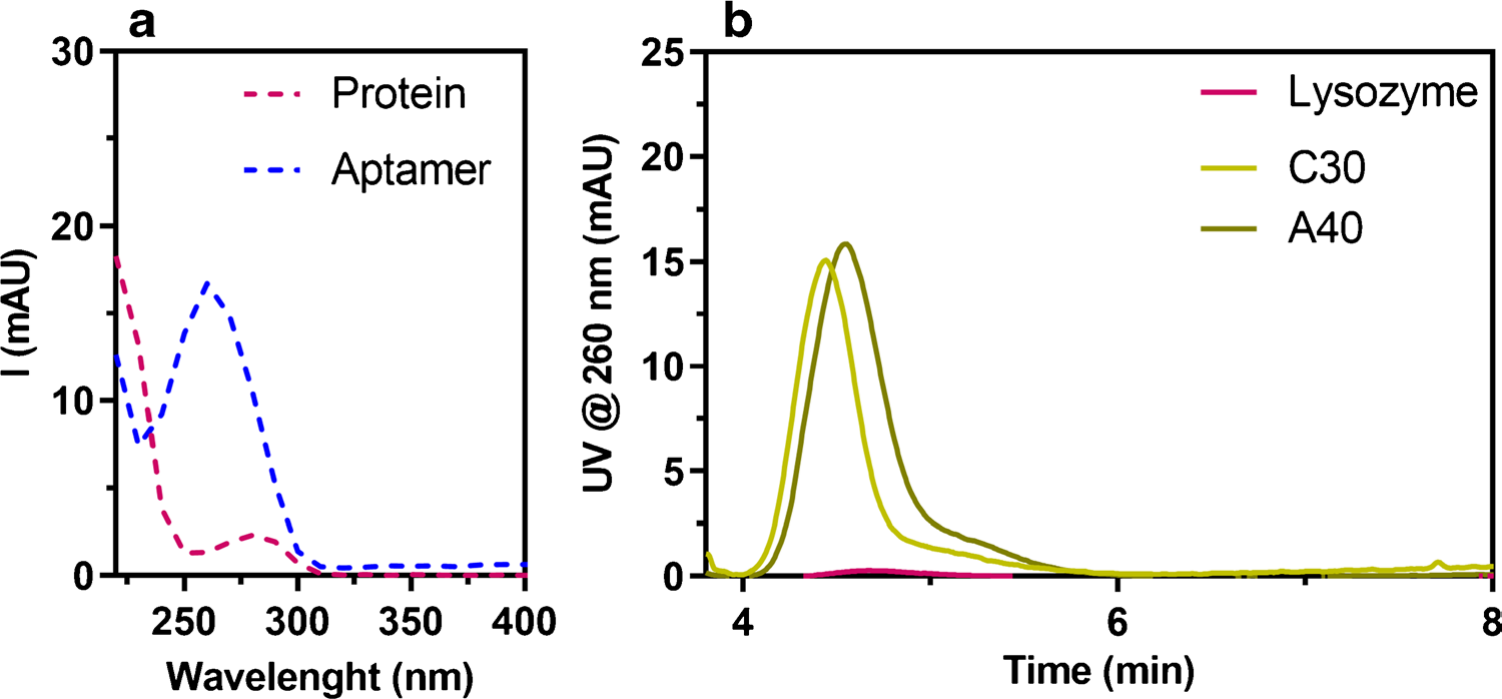
**a** UV spectra of lysozyme as representative protein (pink) and A40 as representative aptamer (blue). **b** AF4-UV overlay fractograms recorded at 260 nm under testing conditions for C30, A40, and lysozyme

It should be noted that the small difference in the retention times of the aptamers and the target lysozyme is a critical issue for the evaluation of their interaction and highlights the need to identify the appropriate parameter to measure and correlate to the formation of a complex. In fact, peak tailing, closeness in molar mass, and band width do not allow for the measurement of the appearance of a new peak corresponding to the complex, which would not be resolved. For these reasons, it was decided to measure the interaction in terms of signal decrease of unbound aptamers, to which a unique signal intensity can be attributed, not influenced by the presence of later eluted species. For the same reasons, we chose to evaluate the intensity decrease at the peak maximum, rather than the signal area. It was also necessary to avoid interference through a strategy based on selective detection of the aptamer. The absorption spectra of lysozyme, BSA, and aptamers, which present the typical relative maxima at 280 nm and 260 nm for proteins and nucleic acids respectively, show that working at 260 nm can selectively monitor the evolution of the aptamer signal (Fig. 2a). This is achievable also thanks to the high absorptivity of aptamers compared to proteins and the low volumes injected, which highlight the difference in signal intensity between the two species.

As starting point for the development of a reliable semi-quantitative approach, LOQ values were calculated for all the aptamers and linearity of aptamers’ signal at 260 nm was also assessed. In particular, LOQs resulted of 4 pmol for C30, 3.4 pmol for C40, and 2 pmol for the 80-mer aptamers; linearity was confirmed between LOQ and the maximum amount injected (40 pmol) for all aptamers ensuring that signal variations could be reliably detected in the envisioned conditions. As for lysozyme, a LOQ of 200 pmol was obtained and its contribution to absorption was therefore negligible in the experimental conditions used (Fig. 2b).

Monitoring the aptamer signal instead of that of the target is also useful to keep the same experimental strategy for the various experimental conditions (i.e., presence of target and potential interfering proteins), for which the screening investigation has to be performed, without the need for further case-by-case optimization. In addition, it should be pointed out that the strength of the present work is the development of a reliable screening strategy for rapid aptamer shortlisting without the need to use tagged oligonucleotides or proteins. In fact, the advantage is the ability to get insights into aptamer-protein interactions for both labeled and not-labeled species likewise.

Moreover, the optimized screening method is able to monitor interactions in one simulated environment in an extremely reduced time: the analysis set relative to the screening of one aptamer in a specific condition is performed in less than an hour. This allowed to perform the entire screening of six aptamers with both lysozyme (target protein) and BSA (interfering agent) within 30 h, including also three replicates for experiments, which highlights the high-throughput of this strategy.

### AF4-UV analysis of aptamer-lysozyme mixtures

**Fig. 3:**
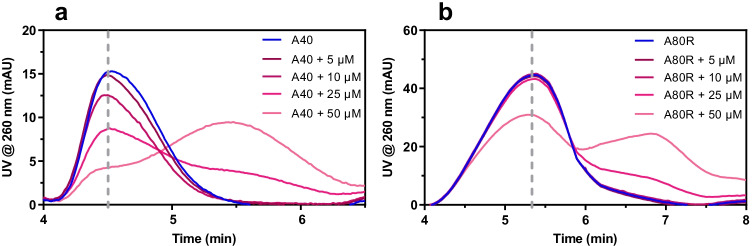
AF4-UV fractograms of **a** A40 and **b** A80R and their mixtures with lysozyme, dashed line: retention time at which signal intensity is recorded to evaluate the signal decrease of free aptamer, correlated to the formation of a complex

As for investigation of the interaction between aptamers and lysozyme, 5 μM of each aptamer was mixed with lysozyme at 5, 10, 25, and 50 μM, and each mix was analyzed with the developed AF4-UV method. The reference value corresponding to the unbound aptamer was recorded by analyzing the aptamer properly mixed with PBS-Mg^2+^ buffer in order to achieve the same concentration of the mixtures. The investigated oligonucleotides showed different interaction profiles, as shown for example in the fractograms for A40 and A80R, reported in Fig. 3. The conformational freedom of unbound aptamers, resulting in a broad AF4 peak, and especially the similar molecular weight of lysozyme and aptamers do not allow for their baseline separation in the fractogram. It was observed that isocratic cross-flow approaches did not improve separation between non-interacting and conjugated aptamer. Despite the absence of baseline separation, it can be observed how the devised strategy is useful to detect and assess the interaction: with the increase in lysozyme concentration, the characteristic signal of the aptamer decreases, while the peak that corresponds to the bound species arises at a retention time compatible to the aptamer-lysozyme complex. On the basis of the recorded fractograms, it is possible to directly visualize the relative binding performances of the aptamers: it is evident that, in the tested medium, A40 presents more affinity to lysozyme respect to A80R. In fact, for A80R, the signal of the complex appears only at high molar excess of the protein in the mixture, while A40 signal decreases with a concentration-dependent trend.

**Fig. 4:**
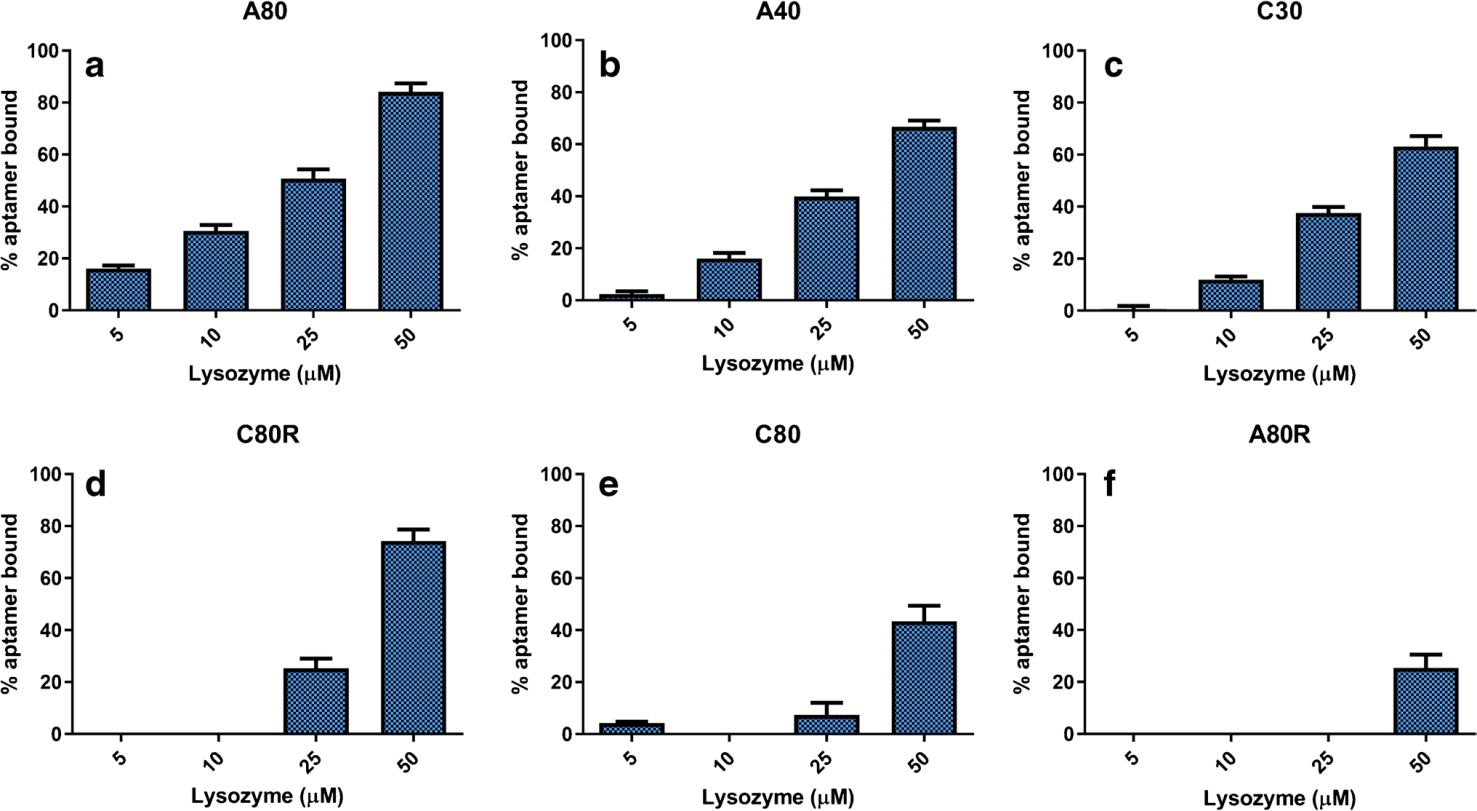
Percentage of bound aptamer (expressed as %(*I*_apt alone_ − *I*_apt mix_)/*I*_apt alone_) for each sequence tested with lysozyme (*n* = 3). **a** A80. **b** A40. **c** C30. **d** C80R. **e** C80. **f** A80R

The fractograms were processed in terms of percentage of bound aptamer respect to the reference (aptamer alone), expressed as %(*I*_apt alone_ − *I*_apt mix_)/*I*_apt alone_. Figure 4 shows the percentage of bound aptamer in function of the different lysozyme concentration levels for all the aptamers investigated: these histograms allow for the visualization and comparison of the performance of each sequence towards the target.

In the concentration and molar ratios investigated, a binding affinity range emerged with A80 (the most interacting sequence) reaching 80% of the bound fraction when mixed to 50 μM of lysozyme, whereas at the same concentration ratio, only 20% of A80R participated in the formation of a complex. The results did not change with a longer incubation time; in addition, it was also noted that no differences in terms of aptamer-protein interaction were observed changing the focus flowrate between 1 and 2.5 mL/min. In general, A80 and A40 showed the highest interaction affinity (Fig. 4a and b), even though it has to be taken into account that the investigated concentrations were at micromolar levels. Lysozyme is also prone to non-specific electrostatic interactions, due to the high isoelectric point (*pI* = 11) and its structure similarity with histones [27]. This can explain why random sequence such as C80R showed an interaction at high molar ratios, even though the high ionic strength of the PBS-Mg^2+^ buffer should contribute to shield the negative charge on DNA and reduce these effects.

### AF4-UV analysis of aptamer-BSA mixtures

BSA was chosen as a representative interfering agent to test specificity, since albumin can be found in different settings as contaminant. For example, BSA could interfere with analyses when lysozyme has to be determined in biological media such as serum, and it is extensively used at high concentration (about 1–2%) as blocking agent in the development of sensing strategies.

**Fig. 5:**
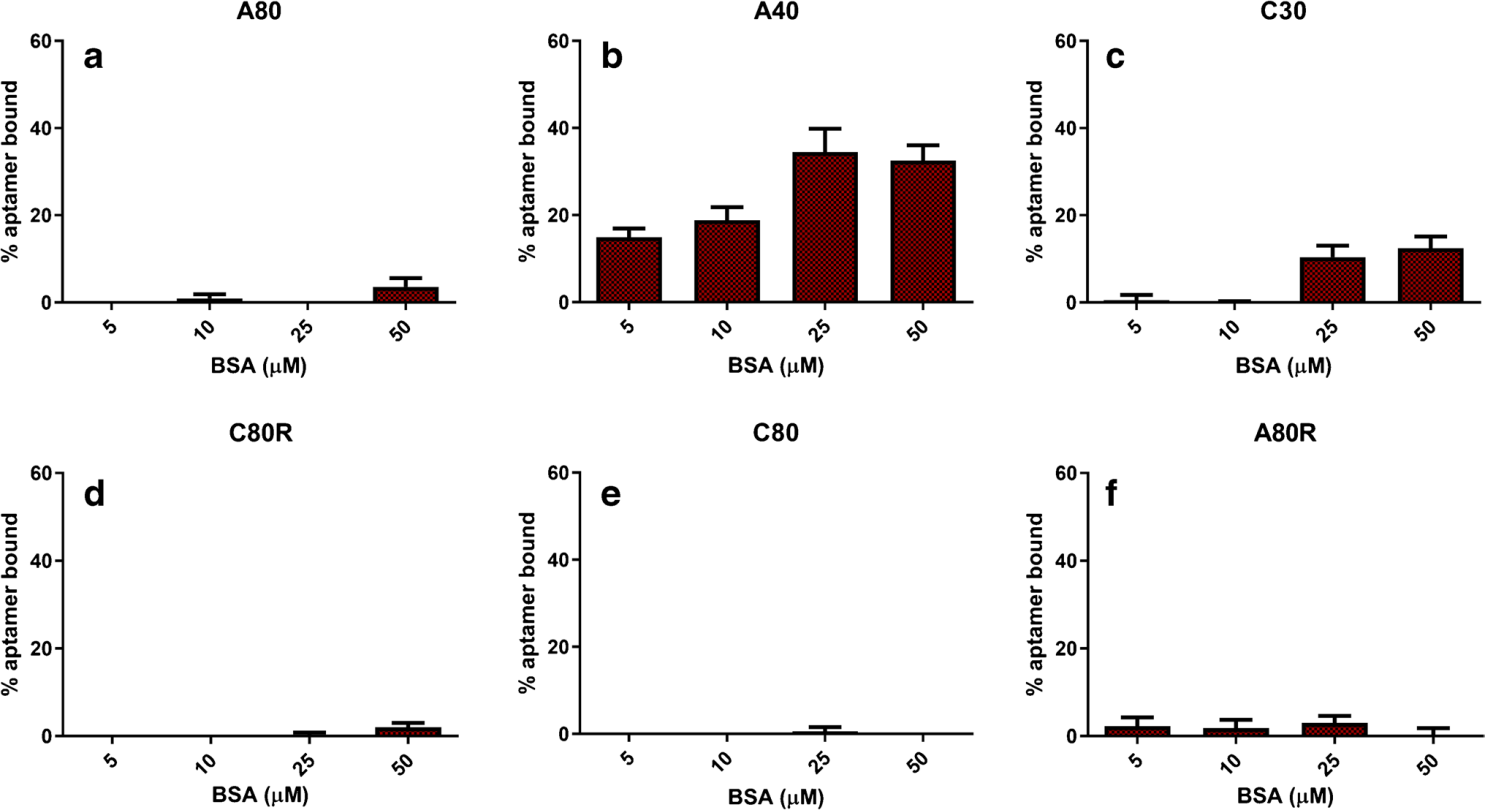
Percentage of bound aptamer (expressed as %(*I*_apt alone_ − *I*_apt mix_)/*I*_apt alone_) for each sequence tested with BSA as representative interfering agent (*n* = 3). **a** A80. **b** A40. **c** C30. **d** C80R. **e** C80. **f** A80R

The same experimental approach was used for incubation of aptamer sequences and BSA at growing concentrations, and the rationalized results are shown in Fig. 5. As examples, AF4-UV fractograms related to the interaction study of A40 and A80 with BSA are shown in Fig. S1 of the Supplementary Information.

It can be observed that the investigated sequences have a much lower affinity towards BSA. However, A40 resulted the most interfered aptamer, reaching 35% of bound fraction. This result is crucial in pointing out that while the priority of SELEX procedure is the selection of an affine aptamer, it is fundamental that the selectivity and cross-reactivity are also tested afterwards.

Combining the obtained results, the potential of this screening proved useful to decide which, in this framework, was the most promising candidate for further development of analytical methods based on aptamers as recognition elements, such as aptasensors. In fact, the affinity of A40 towards lysozyme can be interfered in the presence of BSA, whereas A80 resulted in not interacting with BSA at the concentration levels explored. Furthermore, this same method can be extended to verify the selectivity against other interfering agents or the binding affinity towards other modified targets, in order to assist the development of aptamer-based analytical devices. In fact, the present work represents the first example of AF4-UV characterization of aptamer-lysozyme system, with the overall aim to propose a wide-range application strategy that could also be exploited for the characterization of other aptamer-protein systems, including likewise target and interferent proteins and tagged or not-tagged species.

## Conclusions

The present work demonstrates the potentialities of the AF4-UV technique to develop promising screening methods and get insights into the interaction between aptamers and proteins; in particular, the attention was focused on anti-lysozyme aptamers and randomized sequences. The developed method can be used in the shortlisting of the most performing target-binding aptamers among those previously selected, and carried out in function of downstream analytical applications and relative critical issues, such as possible interfering matrix components. The native conditions in which separation is carried out assure reliability of the obtained results in a particular binding buffer. The approach devised made it possible to directly assess the percentage of bound aptamer in each scenario, by selectively monitoring the aptamer signal decrease, suitable for the assessment of the interaction of the aptamer with the target and with any interfering protein. By comparing the performance of the aptamers against lysozyme and BSA, it was possible to select A80 as the most promising candidate and highlight on the other side the affinity of A40 with both lysozyme and BSA. These results encourage the use of FFF screening downstream SELEX selection and prior to the development aptamer-based devices, since it offers a suitable tool to rapidly evaluate candidate performance and provide additional information on selectivity that can help understand and predict fields of analytical application. In this framework, AF4-UV represents a starting point towards further interaction investigations aimed at providing additional insights into the analytical potentialities of the aptamer sequences.

## Supplementary Information

Below is the link to the electronic supplementary material.

Supplementary file1 (DOCX 141 KB)
