# Supplementary material for: FFF-based high-throughput sequence shortlisting to support the development of aptamer-based analytical strategies
Source: Anal Bioanal Chem. 2022 Feb 18;414(18):5519–27. doi: 10.1007/s00216-022-03971-2 (PMC9242963; doi:10.1007/s00216-022-03971-2)
Supplement: Supplementary file 1 — Supplementary file1 (DOCX 141 KB) [file 216_2022_3971_MOESM1_ESM.docx]

Supplementary Information for:

**FFF-based high-throughput sequence shortlisting to support the development of aptamer-based analytical strategies**

Valentina Marassi^1,2^*, Monica Mattarozzi^3^*, Lorenzo Toma^3^, Stefano Giordani^1^, Luca Ronda^4,5^, Barbara Roda^1,2^, Andrea Zattoni^1,2^, Pierluigi Reschiglian^1,2^, Maria Careri^3^

[valentina.marassi2@unibo.it](mailto:valentina.marassi2@unibo.it); [monica.mattarozzi@unipr.it](mailto:monica.mattarozzi@unipr.it)

^1^ Department of Chemistry, University of Bologna, Via Selmi 2, Bologna, Italy

^2^ byFlow srl, Bologna, Italy

^3^ Department of Chemistry, Life Sciences and Environmental Sustainability, University of Parma, Parco Area delle Scienze 17/A, 43124, Parma, Italy

^4^ Department of Medicine and Surgery, University of Parma, Parco Area delle Scienze, 23/A, 43124, Parma, Italy

^5^ Institute of Biophysics, CNR, 56124 Pisa, Italy


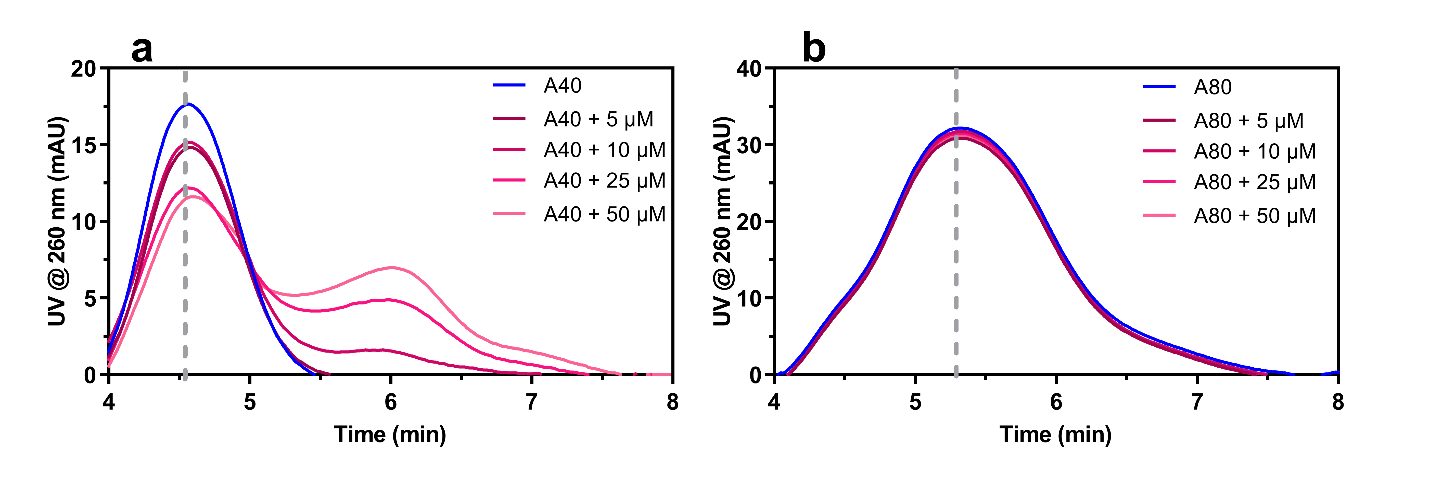


**Fig. S1** AF4-UV fractograms of (a) A40 and (b) A80 and their mixtures with BSA, dashed line: retention time at which signal intensity is recorded to evaluate the signal decrease of free aptamer, correlated to the formation of a complex.
